# Trust in machines: how personality trait shapes static and dynamic trust across different human–machine interaction modalities

**DOI:** 10.3389/fpsyg.2025.1539054

**Published:** 2025-04-29

**Authors:** Yi Zhu, Guiqi Hua, Xinning Liu, Chang Wang, Mingwei Tang

**Affiliations:** ^1^School of Computer Science, Nanjing Audit University, Nanjing, Jiangsu, China; ^2^International School, Nanjing Audit University, Nanjing, Jiangsu, China; ^3^College of Intelligence Science and Technology, National University of Defense Technology, Changsha, Hunan, China; ^4^School of Engineering Audit, Nanjing Audit University, Nanjing, Jiangsu, China

**Keywords:** human–machine interaction, dynamic trust, personality trait, artificial intelligence, interaction modalities

## Abstract

With the rapid advancement of Artificial Intelligence (AI), intelligent machines are increasingly displaying “human-like personality,” shifting Human–Machine Interaction (HMI) from one-way guidance to interactive, multi-level dynamics. Trust plays a pivotal role in the development and maintenance of these evolving interactions. While personality traits have been recognized as key factors in shaping and enhancing interpersonal trust, their role in the development of static and dynamic trust in HMI remains underexplored. This study investigates how personality traits influence trust in HMI across two interaction modalities: Personal Computer (PC) and Virtual Reality (VR). By simulating real-world interaction scenarios, we examined the impact of personality traits on both static and dynamic trust, and explored the relationship between them. The results showed that in the PC modality, personality traits significantly affected both static and dynamic trust, with static trust serving as a strong predictor of dynamic trust. In the VR modality, personality traits significantly influenced static trust, and static trust again played a key role in shaping dynamic trust. These findings underscore the foundational importance of static trust in the evolution of trust in HMI, and highlight the need to consider individual personality differences and interaction modalities in the design of AI-driven interfaces to foster trust and promote long-term engagement.

## 1 Introduction

In recent years, with the rapid advancement of computer technology, Artificial Intelligence has attracted significant public attention. Intelligent machines equipped with advanced algorithms, such as ChatGPT,^1^ ERNIE Bot,^2^ and Qwen,^3^ are increasingly applied in fields like industrial production, education, and daily life, exerting a broad and far-reaching impact (Tian et al., 2024; Berente et al., 2021; Esterwood and Robert, 2023; Gulati et al., 2019; Kulms and Kopp, 2018). Despite their high functionality, these machines still require human guidance and oversight (Xu, 2020; Inkpen et al., 2019; Gulati et al., 2019; Rahwan et al., 2019; Kaber, 2018). When used as productivity tools, intelligent machines can achieve maximum potential through close collaboration with humans (Bansal et al., 2019; Demir et al., 2018). This partnership moves beyond mere mechanical execution, combining subjective human insight with efficient machine computation (Vaccaro and Waldo, 2019). Consequently, the Human-Machine Interaction (HMI) is evolving from one-way guidance to a multi-dimensional, interactive collaboration that encompasses technological, social, cultural, and psychological dimensions.

Continuous technological innovation has enabled intelligent machines to exhibit “human-like” personalities in interactions with humans (Ye and Wang, 2024; Xu and Chao, 2023). Intelligent machines not only communicate with humans but also generate feedback tailored to individual preferences, displaying unique machine personalities that contribute to increasingly complex HMI. As these interactions deepen, the inherent differences between human and machine thinking patterns become evident. Intelligent machines typically rely on binary logic to address problems, distinguishing only between right and wrong. Humans, by contrast, can handle more nuanced possibilities, incorporating logical relationships such as “and,” “or,” and “not” into their decision-making. This divergence in thinking may introduce unpredictable risks in HMI (Xu et al., 2023). For example, human cognitive complexity and emotional biases may add further complexity to interactions with intelligent machines (Conway et al., 2016; Cabitza, 2019). In interpersonal interactions, people often manage uncertainty through trust (Thielmann and Hilbig, 2015), a mechanism that should gradually extend to HMI to help users make appropriate decisions in response to machine outputs. As a result, establishing a trust-based HMI is essential for ensuring collaborative efficiency. Current research on trust in HMI is divided into two main directions: one explores Human-Machine trust theories from a social sciences perspective, focusing on individual factors (Huo et al., 2022; Zhang et al., 2024; Cheng et al., 2022; Lee and See, 2002; Jeon, 2023); the other aims to develop more efficient and accurate AI systems from a computational perspective, emphasizing machine attributes to enhance credibility (Demazure et al., 2021; Vom Brocke et al., 2020; Riedl, 2019).

Among these, personality trait stands out as a fundamental determinant influencing trust, shaping how individuals perceive, respond to, and build trust with intelligent systems (Riedl, 2022; Böckle et al., 2021; Zhou et al., 2020). Despite the significance of these perspectives, they have largely evolved in isolation, leaving a critical gap in understanding how personality trait, as a core individual factor, interacts with machine attributes to form a comprehensive framework for trust in HMI. To address this gap, this paper investigates the pivotal role of personality trait in shaping trust in HMI. By simulating real-world interaction scenarios using diverse testing environments, we collect data on participants’ personality trait and track changes in their trust levels during interactions with intelligent machines. This study demonstrates how personality trait influences trust-building processes, offering theoretical and practical insights into integrating human individuality with machine design to optimize trust in HMI.

## 2 Literature review

### 2.1 The definition of human–machine trust

Trust is understood and interpreted differently across disciplines. In social sciences, trust is seen as a blend of emotion and cognition, emphasizing its fundamental role in fostering social interactions (Lewis, 1985). In psychology, trust is closely tied to individual personality trait, suggesting that personality trait often determines the depth and extent of one’s trust in others (Riedl, 2022). Additionally, psychology view trust as a risk management strategy, helping individuals prepare for potential harm from others’ actions (Dunning et al., 2012). In economics, trust is often viewed as a result of rational decision-making, based on the belief that individuals trust others when it aligns with their self-interest or promotes a collective benefit (Gambetta, 2000). Economists typically see trust as both a product and a well-functioning economic system, playing a key role in enhancing economic efficiency.

Despite these disciplinary differences, all fields recognize the central role of trust in interactive relationships. In interpersonal interactions, both parties are human, and emerges from their differing interests and positions. Trust mitigates this risk by enabling informed judgments under uncertainty, though it also requires the trusting party to accept the possibility of harm from the other’s actions. Similarly, in HMI, trust involves the implicit acceptance of unknown risks. In the context of Human-Machine trust, scholars have offered various definitions. Esterwood et al. (2021) defines it as an individual’s willingness to take risks involving intelligent machines, while Akash et al. (2019) describe it as the extent to which a person relies on such machines. Although these definitions vary in focus, they share a common emphasize on the human intention to trust intelligent machines, reflecting behavioral tendencies toward the machine as the target entity.

With the in-depth study of HMI, some scholars argue that Human-Machine trust should not be solely based on the success rate of a specific task or a particular stage of cognition. Instead, it should be examined within the context of continuous interactions between the trusting parties (Rhim et al., 2023). In interpersonal relationships, trust is gradually established and deepened through ongoing exchanges between cooperating individuals (Rempel et al., 1985). Sociology categorizes trust into two stages: **static trust,** which is based on limited information at the beginning of a relationship, and **dynamic trust**, which evolves through actual interactions over time (Zhu et al., 2023). Drawing from this framework, Human–Machine trust can also be categorized into static and dynamic phases. Static trust develops prior to the establishment of a HMI, when individuals possess limited knowledge and incomplete information about the machine. At this stage, trust judgments are made by assessing perceived risks based on the available information. In contrast, dynamic trust emerges and evolves during ongoing interactions with intelligent machines, shaped by users’ growing understanding and adjusted expectations (Yang et al., 2023; Zhu et al., 2023). This phase is particularly influenced by the outcomes of interactions: positive experiences strengthen trust, while negative outcomes may undermine it, thereby affecting the depth and quality of trust in future collaborations.

A comprehensive understanding of Human–Machine trust must account for both phases, as human trust in intelligent machines evolves through continuous and reciprocal interactions. Hoffman (2017) emphasizes that Human–Machine trust is inherently dynamic—a hybrid process involving human psychological expectations of machine performance and the machine’s actual effectiveness. This dynamic trust is shaped by ongoing, bidirectional exchanges between humans and machines. Consequently, research on Human-Machine trust must address both static and dynamic dimensions to fully capture the complexities of this relationship. Distinguishing between static and dynamic trust provides a novel lens for understanding HMI. Rhim et al. (2023) describe trust as a dynamic parameter that evolves as users interact with intelligent machines. However, current research on Human-Machine trust primarily focuses on static trust, with limited attention given to the mechanisms of dynamic trust. This gap underscores the need for further exploration of how trust develops and adapts over time in HMI.

### 2.2 Influencing factors on human–machine trust

Human–Machine trust is shaped by a combination of factors, which can be broadly categorized into **technical factors, human factors, and contextual factors** (Hancock et al., 2011). These factors work together to influence the level of trust humans place in intelligent machines. From a technological perspective, many studies focus on improving the technical quality and reliability of intelligent machines to enhance mutual trust. The underlying assumption here is that the performance of intelligent machines plays a central role in determining the level of human trust. Key attributes that strengthen trust include reliability, robustness, effectiveness, and understandability. Additionally, clear intent and alignment with moral frameworks contribute to human confidence in machine behavior (Gebru et al., 2022). Moreover, intelligent machines can further solidify this trust by enhancing their dependability, competence, and predictability. Beyond performance, ensuring fairness, interpretability, auditability, and security is crucial for fostering trust (Toreini et al., 2020). These attributes enable users to perceive intelligent machines as trustworthy, safe, and transparent collaborators.

Contextual factors significantly influence trust in HMI. Factors such as the educational system, technology acceptance, and regional cultural values create a complex interaction context that shapes how individuals engage with intelligent machines. In specific cultural settings, individuals may exhibit stronger trust in intelligent systems when faced with complex tasks that exceed their cognitive capacity (Ahmad et al., 2021). The characteristics of the task itself are also crucial: as task demands and workload increase, the likelihood of errors rises, often reducing trust as individuals opt to complete tasks independently (Esterwood and Robert, 2023). Conversely, tasks that surpass an individual’s processing capacity may lead to greater reliance on intelligent machines, which can impact self-perception, psychological stability, and trust. For instance, visual search tasks nearing cognitive limits tend to foster increased trust in intelligent systems (Driggs and Vangsness, 2022). These findings suggest that task difficulty interacts with individual personality trait and contextual factors to shape trust dynamics.

Moreover, the interaction modality used for HMI is a central factor influencing trust in the establishment of HMI. Different interaction modalities can result in varying levels of trust in intelligent machines. For instance, traditional personal computer (PC) modalities are characterized by structured, predictable interactions through 2D graphical interfaces and standard input devices like keyboards and mice. While this modality is familiar and reliable, its limited sensory engagement and interactivity can hinder the development of dynamic trust, particularly in tasks requiring high adaptability or realism. Users in such contexts may find it harder to assess machine performance intuitively, which can reduce trust in complex scenarios (Peng and Ke, 2015; Chen J. et al., 2024). In contrast, virtual reality (VR) modalities offer immersive and interactive settings that engage users more deeply. VR has been widely adopted in fields such as education, healthcare, and communication, where its ability to simulate realistic environments has transformed traditional practices. For instance, in education, VR allows students to explore historical sites, simulate science experiments, and interact dynamically with teachers, enhancing engagement and experiential learning (Familoni and Onyebuchi, 2024). Similarly, in healthcare, VR-based training environments provide realistic simulations for surgical procedures and anatomy teaching, leading to improved task accuracy and skill acquisition (Combalia et al., 2024). Although studies show that VR leads to improvements in surgical time, task accuracy, and cost-effectiveness compared to PC-based (Mao et al., 2021), VR’s immersive nature can also introduce challenges, such as cognitive overload, motion sickness, or mental fatigue, which may negatively impact trust in intelligent systems (Vlahovic et al., 2024). The continuous advancement of artificial intelligence has positioned VR as a focal point for HMI research, due to its potential to increase trust through enhanced interactivity and sensory immersion (Keighrey et al., 2020; Partarakis and Zabulis, 2024). At the same time, the distinct characteristics of traditional and immersive modalities highlight the importance of contextual factors in shaping Human–Machine trust. Consequently, the study of trust across these different modalities requires further exploration. To investigate these dynamics, this study compares trust in HMI across two distinct modalities: traditional PC-based and immersive VR-based contexts.

In addition to contextual and technical factors, human factors play a profound role in shaping Human-Machine trust (Matthews et al., 2021; Fisher et al., 2012). Haidt (2012) argued that the establishment of interpersonal trust is influenced by six individual attributes: caring, freedom, fairness, loyalty, power, and kindness. These attributes are shaped by factors such as age, personality, past experiences, and cultural background. Together, they encompass not only biological aspects, such as cognitive abilities and emotional states, but also aspects of socialization, including educational background and cultural experiences. As a result, trust in intelligent machines can vary significantly between individuals, even when interacting within similar technological conditions and task contexts. For instance, Lee et al. (2021) demonstrated that familiarity with artificial intelligence is positively correlated with trust, suggesting that an individual’s exposure to and experience with technology plays a critical role in shaping their level of trust in intelligent systems. Despite extensive research on how technical and contextual factors influence Human-Machine trust, the role of human factors, particularly personality trait, remains underexplored.

### 2.3 Personality trait and trust

Personality trait plays a central role in the establishment of trust, particularly in HMI, where its influence is both significant and multifaceted (Riedl, 2022). Research on personality trait has developed a robust theoretical framework, drawing insights from disciplines such as psychology, management, and sociology. These studies emphasize the crucial role of personality trait in shaping diverse phenomena and influencing outcomes, highlighting its significance in understanding and explaining individual behavioral and emotional responses.

In psychology, personality trait is regarded as the stable foundation of an individual’s behavioral and emotional tendencies (Funder, 2001). High levels of positive personality trait, often referred to as “personality strengths,” represent attributes that enhance an individual’s ability to adapt, manage stress, and achieve goals (Dametto and Noronha, 2021). These strengths act as a form of psychological capital, promoting resilience, harmonious relationships, and improved life satisfaction while alleviating psychological stress. In the context of mental health, personality strengths serve as “psychological immunity,” enabling individuals to cope with adverse life events and emotional challenges (Park and Peterson, 2006). For instance, college students with personality trait like resilience, optimism, or self-efficacy are better equipped to navigate stressful situations, such as academic pressures and interpersonal conflicts, fostering greater emotional well-being and adaptability (Karris Bachik et al., 2021).

In management science, personality trait is recognized as the cornerstone of interpersonal interactions, profoundly shaping how individuals respond to others and approach various challenges. Personality trait is formed through a combination of genetic predispositions and environmental influences (Park and Peterson, 2009). It plays a pivotal role in interpersonal communication, cognitive development, skill enhancement, and productivity improvement (Noronha and Campos, 2018; Therasa and Vijayabanu, 2015). In similar environments or under comparable challenges, different personality trait lead individuals to adopt varying behaviors and strategies. These personality trait influences not only an individual’s perception of the external world but also their problem-solving and decision-making processes. In organizational contexts, the personality trait of leaders impacts the trust atmosphere within an organization and is closely tied to its operations and performance. When a leader’s personality trait aligns well with their roles and responsibilities, organizational trust tends to be higher, and leadership effectiveness improves, fostering innovation and operational efficiency. Conversely, mismatches between personality trait and responsibilities can erode trust, trigger internal conflicts, and impair organizational functioning. To optimize performance, leaders and teams must align personality trait with roles and adopt advanced management strategies to build trust and enhance collaboration (Ahmad et al., 2021; Simic et al., 2022; O’Neil, 2007).

In sociology, interpersonal trust is essential for effective communication, with personality trait playing a vital role in the formation and persistence of trust. Research highlights that certain personality traits are more particularly conducive to establishing and maintaining trust, aligning with the Big Five personality theory in psychology (Gosling et al., 2003). This framework categorizes personality traits into five core dimensions: Conscientiousness, Openness to Experience, Agreeableness, Emotional stability, and Extraversion (Roos and Kazemi, 2022). For instance, individuals with high levels of Conscientiousness and Openness to Experience are more likely to develop strong trusting relationships, while Agreeableness is particularly linked to the formation of trust with strangers (Freitag and Bauer, 2016). As interactions deepen, these personality traits continue to shape how individuals develop and sustain trust, influencing both interpersonal and broader social relationships.

The influence of personality trait on trust between humans and intelligent machines is receiving growing attention, particularly in HMI. Establishing and maintaining trust in these settings has emerged as a critical research focus (Jacovi et al., 2021; Matthews et al., 2021; Böckle et al., 2021). Research on personality trait in HMI can be categorized into two primary areas: first, studies have explored how individual personality traits affect trust in machines. Personality traits such as Openness to Experience, Extraversion, and Emotional stability have been found to have a direct relationship with trust in intelligent systems (Riedl, 2022; Böckle et al., 2021). These personality traits are particularly influential in HMI scenarios involving high levels of uncertainty and cognitive load, where trust becomes a crucial factor for effective interaction (Zhou et al., 2020). Second, research has examined how intelligent machines can influence human trust by mimicking “human-like personality.” Machines achieve this by employing vocal and linguistic features, as well as non-verbal cues such as gestures and visual textures. For instance, studies have shown that intelligent machines mimicking extroverted personality traits through vocal and linguistic features are generally more trusted and preferred than those mimicking introverted personality traits (Lim et al., 2022). Moreover, non-verbal cues designed to convey “human-like personality” not only enhance perceptions of the machine’s social presence but also influence levels of trust in HMI (Chien et al., 2022).

In summary, research on Human-Machine trust has examined both how individual personality traits influence trust in intelligent machines and how intelligent machines shape human trust behaviors by simulating human-like personality. However, much of the existing work has focused on trust established during initial HMI (i.e., static trust), with relatively fewer studies have examined dynamic trust, i.e., the trust that evolves as interactions deepen over time. This paper integrates trust theories from psychology to investigate how personality trait, along with contextual factors such as interaction modalities, influences the establishment of Human-Machine trust, with a particular focus on dynamic trust development.

Based on the research discussed above, trust is a dynamic parameter that evolves through continuous two-way interaction between humans and intelligent machines. Human-Machine trust is built gradually, making it essential to examine both static and dynamic trust in studies of this phenomenon. Among contextual factors influencing Human-Machine trust, interaction modalities play a crucial roles, and differences in how these factors affect static and dynamic trust warrant further investigation. Regarding human factors, personality trait is a key determinant of Human-Machine trust. However, most existing research on personality trait focuses on static trust, leaving its role in dynamic trust and its interaction with contextual factors unclear and in need of further exploration. In light of these considerations, the following hypotheses are proposed:

*H1*: Personality trait has a significant effect on static trust in HMI across varying interaction modalities.

*H2*: Personality trait has a significant effect on dynamic trust in HMI across varying interaction modalities.

*H3*: Static trust has a significant effect on dynamic trust in HMI, moderated by interaction modalities.

## 3 Materials and methods

### 3.1 Experimental design

To investigate the influence of personality trait on Human-Machine trust, this study developed an experimental software platform within the Unity environment to simulate a drone surveillance mission. The choice of a done (also known as Unmanned Aerial Vehicles, UAV) surveillance mission is well-suited for studying Human-Machine trust for several reasons. UAVs are highly automated, intelligent systems that require close collaboration with human operators, especially in high-risk or critical missions. Trust is a critical factor in determining the effectiveness of such collaborations, making UAVs an ideal subject for examining trust dynamics. Furthermore, UAV operations involve complex tasks such as spatial perception, decision-making, and coordination, which are likely to be influenced by the operator’s personality traits. This provides a rich context for exploring the role of personality trait in HMI. Additionally, UAVs equipped with AI technologies can simulate “human-like personality,” offering an opportunity to study how such behaviors influence trust development.

In this study, the path planning of UAVs are achieved through a deep learning-based approach, utilizing the Double Deep Q-Network, i.e., D3QN, algorithm (Chang et al., 2020) to optimize navigation in dynamic environments, particularly when faced with enemy threats such as radar detection. This algorithm enables UAVs to efficiently perform obstacle avoidance and strategic maneuvering by leveraging a well-structured neural network. The fundamental principles of the D3QN algorithm can be outlined as follows:


$$y^{j}=r^{j}+\gamma Q\left(s^{j+1},\underset{a}{\arg\;\max}Q\left(a^{j+1},a;\theta \right);\theta^{-}\right)$$

Where “r” represents the reward function value; “s” represents the system state; “θ^−^” represents the target network parameters, “θ” represents the main network parameters, “a” represents the roll action, and “γ” represents the discount factor. The D3QN architecture consists of two primary components: a Multilayer Perceptron, i.e., MLP, and a dueling network, both designed to process UAV state-action interactions and enhance decision-making. The MLP comprises three fully connected layers with 64, 256, and 128 hidden nodes, each employing the Rectified Linear Unit, i.e., ReLU, activation function to ensure efficient learning and gradient propagation. Meanwhile, the dueling network is divided into two branches: the state-value function and the advantage function, both featuring an initial 64-node fully connected layer. The state-value function branch further includes a second layer with a single node, responsible for outputting the overall value of the UAV’s current state, whereas the advantage function branch contains three nodes, estimating the relative benefit of each possible action. By integrating these components, the D3QN algorithm significantly enhances UAV autonomy, allowing for adaptive, intelligent navigation while mitigating potential threats in adversarial environments.

The experimental setup consists of one manned aerial vehicle (MAV) and four UAVs, each utilizing deep reinforcement learning algorithms for path planning, obstacle avoidance, and flight control. This design enables an in-depth investigation of how personality trait and contextual factors influence the development of trust across multiple dimensions, including static and dynamic trust. The experiment takes place in a virtual environment featuring multiple detection points. Each detection point is represented as a rectangular box, and the drones are required to detect these points. Upon successful detection, the box changes color, signaling task completion. To introduce complexity and risk, radar systems continuously monitor the surrounding airspace for unauthorized objects in designated “danger zones.” If a UAV enters a danger zone for a certain duration, it is deemed “exposed,” resulting in mission failure. Participants control the MAV, guiding it from the starting point through all detection points and ensuring it ultimately reaches the endpoint. If the MAV approaches or enters a danger zone, an alarm is triggered, requiring participants to make adjustments to prevent mission failure. The goal of the experiment is to complete the mission successfully by detecting all points, avoiding danger zones, and reaching the endpoint. To control experimental variables, the UAVs are equipped solely with surveillance functions, with all path planning and obstacle avoidance handled autonomously using deep reinforcement learning. This ensures that participants focus on collaboration rather than direct UAV control, isolating trust-related factors for analysis.

The experimental process was divided into two phases: *pre-interaction* and *interaction*. This structure facilitated the measurement of both static and dynamic trust while maintaining control over experimental conditions.

#### 3.1.1 Pre-interaction phase

During the pre-interaction phase, participants were introduced to the experimental objectives and procedures. They completed standardized personality trait questionnaire to assess individual personality traits. The instructor then provided an overview of the AI algorithms used in the UAVs to ensure participants understood their functionalities and capabilities. Participants then engaged in a training session, piloting the MAV to complete a single-point detection mission. This training familiarized participants with the experimental setup, ensuring a clear and consistent understanding of the task. Upon completing the training, participants were asked to report their static trust levels (T_0_).

#### 3.1.2 Interaction phase

In the post-interaction phase, participants collaborated with the UAVs to complete the full surveillance mission. This involved detecting all points, avoiding danger zones, and successfully guiding the MAV to the endpoint. Upon completing the task, participants were asked again to report their trust levels (T_1_). By comparing the data collected in the pre-interaction and interaction phases, we aimed to analyze how trust evolved during the interaction and how personality trait and contextual factors influenced this process.

### 3.2 Interaction modalities

To explore the influence of personality trait on Human-Machine trust across different contexts, the experiment was conducted in two interaction modalities: personal computer (PC) and virtual reality (VR). Both modalities were designed to ensure consistency in the experimental setup while varying sensory and interaction dynamics. In the PC modality, participants interacted with the simulation using standard input devices, such as a keyboard and mouse, while seated at a monitor. In the VR modality, participants used a VR headset to engage with an immersive version of the same simulation. These two modalities enabled an examination of how sensory engagement and cognitive load interact with personality trait to influence the development of trust.

#### 3.2.1 PC modality

**Figure 1:**
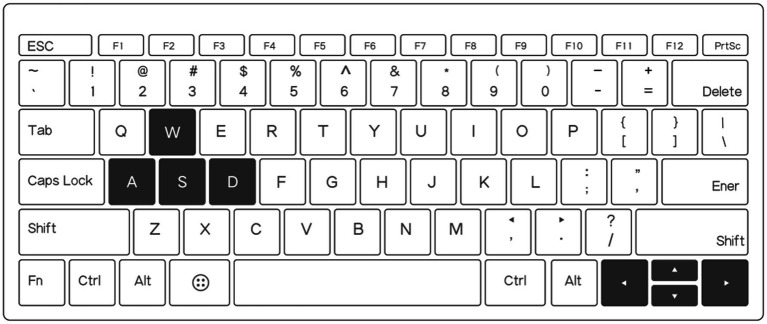
Schematic diagram of the keypad, the black keys are used to control the roll angle and pitch angle of the MAV.

The experimental equipment for the PC modality consisted of a computer running Windows 10, a 24-inch monitor with a 144 Hz refresh rate and a resolution of 2048 × 1,080, and external input devices, including a mouse and keyboard. As shown in Figure 1, participants used the keyboard to control the flight direction of the MAV. Specifically, the “W” and “S” keys control the pitch angle (up and down), while the “A” and “D” keys control the roll angle (left and right) of the MAV. The up, down, left, and right arrow keys on the keyboard correspond to the same functions as the “W,” “S,” “A,” and “D” keys. Participants could choose either control method to operate the MAV.

**Figure 2:**
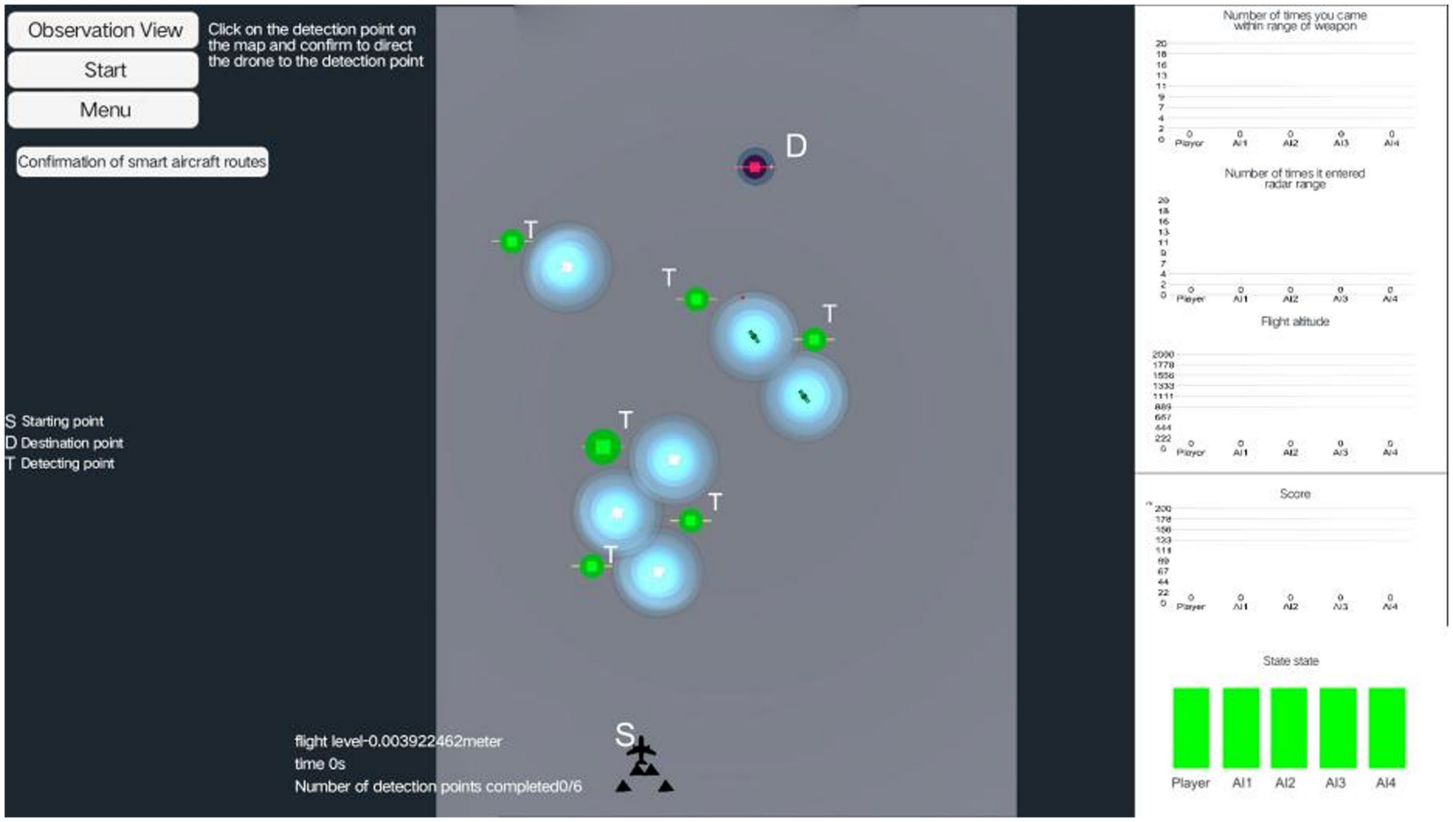
Panoramic view of the PC experiment scene, the left side indicates various operation buttons, the center indicates the specific scene of the experiment, and the right side indicates the current real-time data of the MAV and the UAV.

The experimental screen setup was illustrated in Figure 2. In this setup, “S” represented the MAV’s starting point, “D” represented the destination, “T” represented the detection points, and the blue area near each detection point indicated the danger zone. The primary objective of the experiment was to start at “S,” detect all “T” points while avoiding the danger zones, and finally reached “D.” During the detection task, real-time data for the MAV—including altitude, time, and the number of detected targets—were displayed at the bottom of the screen, while real-time data for the UAVs appeared on the right side. The MAV under test can collaborate with the UAVs to accelerate task completion by jointly detecting all designated points. If the participant wanted the UAVs to detect a specific point, they can click on that detection point with the mouse, which would display the “Confirm Intelligent Route” button on the right side of the screen. By clicking this button, the UAVs would automatically proceed to detect the selected point.

#### 3.2.2 VR modality

**Figure 3:**
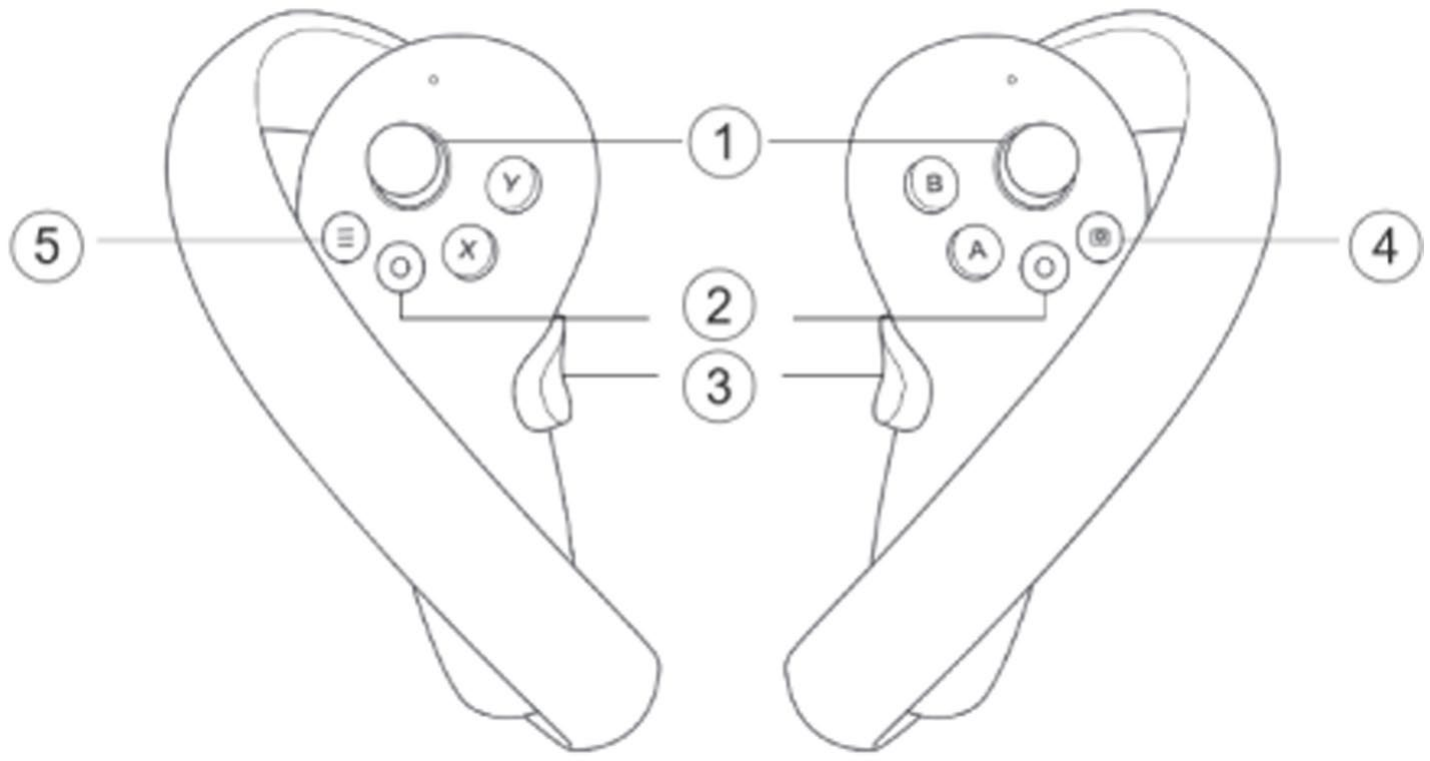
Schematic diagram of the operation of the VR end handle, 1 is the joystick, 2 is the Home button, 3 is the grip button, 4 is the cutoff button, 5 is the menu button.

The VR setup for the experiment included Pico 4 VR glasses,^1^ offering a resolution of 4320 × 2160 and a 90 Hz refresh rate, along with two handheld controllers to ensure an immersive and high-quality experience for participants. The controllers were designed for intuitive navigation and control of the MAV within the virtual environment. The primary joystick (labeled ‘1’ in Figure 3) controlled both the speed and direction of the MAV, providing precise maneuverability. Additional buttons included the Home button (‘2’), which returned participants to the home screen, and the Grip button (‘3’), which activated or recalled the cursor. The Cutoff button (‘4’) allowed participants to take screenshots of the current view, while the Menu button (‘5’) provided access to additional settings and options. This setup was intended to enhance the experiment by allowing realistic, hands-on control over the MAV, enabling participants to effectively engage with the simulated mission environment.

**Figure 4:**
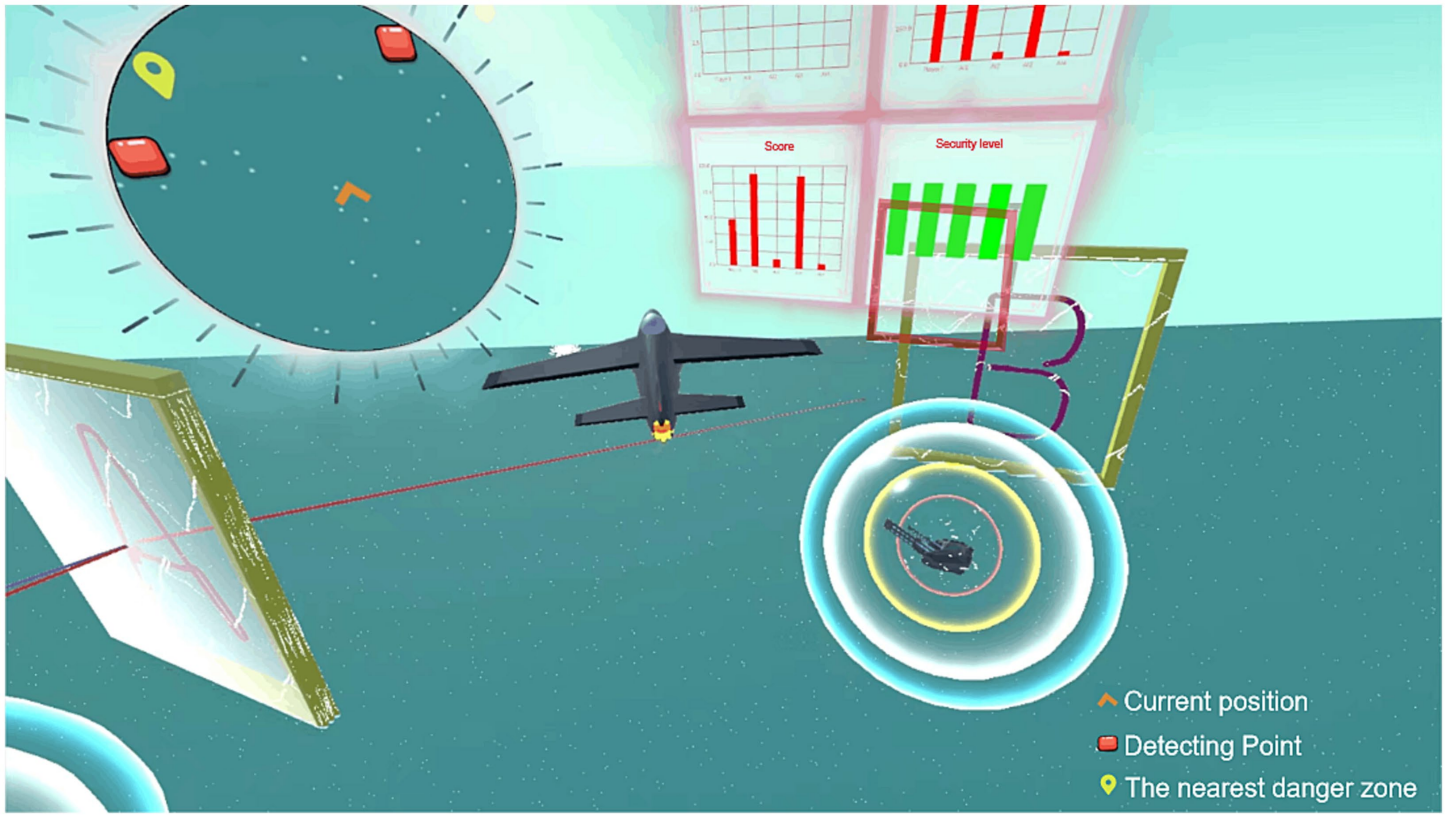
VR experiment scene interface, the upper left circle indicates the panoramic view, the main interface indicates the flight perspective of the MAV, and the middle of the interface indicates the real-time data of the current flight.

The detailed interface layout of the VR experimental environment was shown in Figure 4. The main interface displayed multiple detection points and danger zones scattered throughout the environment, providing a dynamic landscape for the mission. In the center of the interface, a dashboard provided real-time data such as the current flight altitude, score, safety status, and the number of times the MAV or UAV has entered the radar range. Participants begin the task at a starting point, navigating both the MAV and UAVs to the green rectangular detection points, labeled “A” and “B,” that need to be covered. The goal was to complete all detection points and guided the MAV and UAVs to the red-marked destination. As the mission progresses, warnings were triggered if the MAV or UAVs approached a danger zone, and the mission may be terminated if the vehicles entered the core of the danger zone. The top left corner of the interface featured a mini-map, where the orange symbol represented the MAV’s current position, the red squares marked the detection points, and the yellow landmarks indicated the nearest danger zones. If the participant wished for the UAVs to detect a specific detection point, they can use the joystick to select the corresponding button for that point. Upon clicking, the UAVs would automatically proceed to detect the point, facilitating collaboration in the mission.

### 3.3 Measurements

This study employs the Big Five theory of personality, also known as the “Big Five,” to measure personality trait. The Big Five is a prominent theory in psychology used to describe and categorize personality traits (Riedl, 2022). According to this theory, an individual’s personality trait is categorized into five main dimensions:

#### 3.3.1 Openness to experiences

This dimension measures an individual’s receptivity to new experiences and willingness to explore. Individuals high in Openness to experiences tend to be more innovative, curious about novelty, and sensitive to art and culture. In contrast, those with low Openness to experiences are typically more conservative and traditional.

#### 3.3.2 Conscientiousness

This dimension measures an individual’s sense of responsibility, organizational skills, and goal orientation. Individuals with high Conscientiousness typically demonstrate strong organizational skills, reliability, and self-discipline. In contrast, those with low Conscientiousness tend to be more spontaneous or careless with details.

#### 3.3.3 Extraversion

This dimension measures an individual’s activity level in social and external environments. Individuals with high Extraversion tend to be outgoing, talkative, and enjoy socializing with others. In contrast, individuals with low Extraversion are often more introverted and independent.

#### 3.3.4 Agreeableness

This dimension measures an individual’s friendliness, cooperation, and empathy in interpersonal interactions. Individuals high in Agreeableness tend to be kind, compassionate, and cooperative. In contrast, individuals with low Agreeableness may display more critical, antagonistic, or apathetic behavior.

#### 3.3.5 Emotional stability

This dimension measures Emotional stability and the ability to manage negative emotions. Individuals with high Emotional stability are less affected by strong negative emotions and tend to remain calm under pressure. In contrast, individuals with low Emotional stability may be more prone to experiencing emotions such as anxiety, depression, or anger.

The Big Five personality theory serves as the foundation for describing individual personality trait and has been widely applied in fields such as mental health and career choice (Riedl, 2022). To systematically assess individual personality trait, the Chinese version of the Ten-Item Personality Inventory (TIPI) (Gosling et al., 2003) was used as the measurement tool in this experiment. The questionnaire consists of 10 items, as shown in Table 1, each corresponding to one of the five personality trait dimensions described above. Each dimension is measured by two opposing questions; for example, the Extraversion dimension includes question 1 (“extroverted, enthusiastic”) and question 6 (“reserved, quiet”). The questionnaire employs a seven-point Likert scale, with responses ranging from 1 (strongly disagree) to 7 (strongly agree). The scores for each dimension were calculated by adding up the scores of the corresponding items and then averaging them, as shown in the formula below:


$$\mathrm{Extraversion}=Q1+\left(8-Q6\right)$$

$$\mathrm{Agreeableness}=Q7+\left(8-Q2\right)$$

$$\mathrm{Conscientiousness}=Q3+\left(8-Q8\right)$$

$$\mathrm{Emotional}\;\mathrm{stability}=Q9+\left(8-Q4\right)$$

$$\mathrm{Openness}\;to\;\mathrm{experiences}=Q5+\left(8-Q10\right)$$

**Table 1:** The 10-item personality inventory.

I see myself as:	1	2	3	4	5	6	7
Q1: Extraverted, enthusiastic.	○	○	○	○	○	○	○
Q2: Critical, quarrelsome.	○	○	○	○	○	○	○
Q3: Dependable, self-disciplined.	○	○	○	○	○	○	○
Q4: Anxious, easily upset.	○	○	○	○	○	○	○
Q5: Open to new experiences, complex.	○	○	○	○	○	○	○
Q6: Reserved, quiet.	○	○	○	○	○	○	○
Q7: Sympathetic, warm.	○	○	○	○	○	○	○
Q8: Disorganized, careless.	○	○	○	○	○	○	○
Q9: Calm, emotionally stable.	○	○	○	○	○	○	○
Q10: Conventional, uncreative.	○	○	○	○	○	○	○

1 = Disagree strongly; 2 = Disagree moderately; 3 = Disagree a little; 4 = Neither agree nor disagree; 5 = Agree a little; 6 = Agree moderately; 7 = Agree strongly.

In addition, the study assessed participants’ trust levels by asking the question, “How much do you trust the UAV with AI algorithms?.” And the study assessed participants’ knowledge of AI by asking the question, “The level of understanding of AI?.” Participants responded using a seven-point Likert scale, ranging from 1 (“Not at all”) to 7 (“Completely trustworthy”).

## 4 Results

### 4.1 PC experiment

The PC experiment involved 80 participants from diverse disciplinary backgrounds. Of these, 22.5% were male and 77.5% were female. Regarding educational background, 80% of the participants were undergraduates, while 20% were postgraduates. When it came to AI knowledge, 67.5% of participants indicated some familiarity with AI, and 32.5% had limited knowledge. Where a level of understanding greater than 4 is considered some familiarity with AI.

To enhance data analysis, the Box-Cox transformation was applied to preprocess the dataset (Osborne, 2010; Zhang and Yang, 2017). The Box-Cox transformation is defined as follows:


$$y^{\left(\lambda \right)}=\left\{\begin{array}{c} \frac{y^{\lambda }-1}{\lambda },\lambda \neq 0\\ \ln\left(y\right),\lambda =0 \end{array}\right.$$

where “y^(λ)^” represents the transformed data, “y” is the original data, and “λ” is the transformation parameter. Specifically, the λ values for different variables are as follows: 1.068 for Extraversion, 1.786 for Agreeableness, 0.903 for Conscientiousness, 1.318 for Emotional stability, 1.508 for Openness to experiences, 0.601 for T_0_, and 1.776 for T_1_. The skewness and kurtosis of the transformed data are presented in Table 2, where the absolute values of both coefficients are less than 1.96, indicating no significant deviation from zero. This confirms that the transformed data follows a normal distribution, ensuring its suitability for further statistical analysis.

**Table 2:** Kurtosis and skewness of PC data.

	Kurtosis	Kurtosis coefficient	Skewness	Skewness coefficient
1. Extraversion	−0.024	−0.089	−0.031	−0.058
2. Agreeableness	−0.123	−0.457	−0.863	−1.622
3. Conscientiousness	0.052	0.193	0.766	1.440
4. Emotional stability	−0.021	−0.078	0.166	0.312
5. Openness to experiences	−0.089	−0.331	−0.448	−0.842
6. T_0_	−0.014	−0.052	−0.341	−0.641
7. T_1_	−0.038	−0.141	−0.126	−0.237

To explore the relationships between personality traits and trust levels, Pearson correlation analysis was conducted. This method was chosen because it is well-suited for identifying linear associations between variables, providing initial evidence for the hypothesized relationships between personality trait and trust. The results are presented in Table 3 and show significant positive correlations. Specifically, Extraversion was positively correlated with static trust T_0_ (*r* = 0.315, *p* < 0.01), while Emotional stability was positively correlated with trust after interaction T_1_ (*r* = 0.286, *p* < 0.05). These findings suggest that higher levels of Extraversion and Emotional stability are associated with greater static and dynamic trust, respectively.

**Table 3:** Pearson correlation analysis.

	*M*	SD	1	2	3	4	5	6
1. Extraversion	3.549	1.258						
2. Agreeableness	10.482	3.652	0.124					
3. Conscientiousness	3.187	0.787	−0.023	0.191				
4. Emotional stability	4.640	1.725	−0.136	0.182	0.409^***^			
5. Openness to experiences	7.035	2.841	0.303^**^	0.294^**^	0.052	0.109		
6. T_0_	2.557	0.460	0.315^*^	−0.053	0.054	−0.071	0.163	
7. T_1_	9.686	3.319	0.186	−0.077	0.107	0.286^*^	0.156	0.350^***^

*p < 0.05, **p < 0.01, ***p < 0.001.

Stepwise regression analysis was employed to identify the most significant predictors of trust while controlling for potential multicollinearity among personality traits and other variables. This approach allows the inclusion of predictors in a systematic manner, retaining only those variables that contribute meaningfully to the variance in the dependent variable. The method is particularly suitable for this study because it provides a clear understanding of the unique contributions of individual personality traits and static trust T_0_ to dynamic trust T_1_. Moreover, diagnostic checks, including Variance Inflation Factor (VIF) values and the Durbin-Watson statistic, were applied to ensure the validity of the regression model and its assumptions. Stepwise regression was first conducted to explore the predictive role of personality traits on static trust T_0_. The results are shown in Table 4. All Variance Inflation Factor (VIF) values were below 10, and the Durbin-Watson value was 1.977, indicating that there is no autocorrelation in the residuals. The regression model demonstrated research significance (*F* = 8.607, *p* < 0.05). The results revealed that Extraversion positively predicted T_0_ (*B* = 0.115, *p* < 0.05), and explained 9.9% of the variance in the dependent variable T_0_ (*R*^2^ = 0.099). These results align with H1, demonstrating that personality traits, particularly Extraversion, play a critical role in shaping static trust in HMI.

**Table 4:** Effect of personality traits on T_0_.

	*B*	*β*	Sig	VIF	*F*	Model sig	*R* ^2^	Durbin-Watson
Extraversion	0.115	0.315	0.004^**^	1.000	8.607	0.004^**^	0.099	1.977
Exclude variables								
Agreeableness		−0.094	0.389	1.016				
Conscientiousness		0.061	0.571	1.001				
Emotional stability		−0.029	0.793	1.019				
Openness to experiences		0.075	0.512	1.101				

*p < 0.05, **p < 0.01, ***p < 0.001.

To examine the factors influencing trust after interaction T_1_, the five dimensions of personality trait, along with T_0_ and T_1_, were analyzed using stepwise regression, and the results are presented in Table 5. The VIF values were less than 10, and the Durbin-Watson value was 1.836, indicating that there is no autocorrelation in the data. The model was found to be statistically significant (*F* = 10.817, *p* < 0.001). Both T_0_ (*B* = 2.685, *p* < 0.001) and Emotional stability (*B* = 0.600, *p* < 0.01) were found to positively predict T_1_. The combination of T_0_ and Emotional stability explained 21.9% of the variance in T_1_ (*R*^2^ = 0.219). These findings support H2 and H3, demonstrating that static trust T_0_ serves as a foundation for dynamic trust T_1_ and that Emotional stability contributes significantly to trust development over time.

**Table 5:** Personality traits and the effect of T_0_ on T_1_.

	*B*	*β*	Sig	VIF	*F*	Model sig	*R* ^2^	Durbin-Watson
T_0_	2.685	0.372	0.000^***^	1.005	10.817	0.000^***^	0.219	1.836
Emotional stability	0.600	0.312	0.003^**^	1.005				
Exclude variables								
Extraversion		0.126	0.236	1.127				
Agreeableness		−0.118	0.280	1.036				
Conscientiousness		−0.050	0.607	1.211				
Openness to experiences		0.064	0.617	1.043				

*p < 0.05, **p < 0.01, ***p < 0.001.

### 4.2 VR experiment

The VR experiment involved 87 participants from diverse disciplinary backgrounds. The gender distribution consisted of 52.8% male and 47.1% female participants. Educational background varied, with 27.6% being undergraduates and 72.4% being graduate students. Regarding familiarity with AI, 73.6% of participants reported having some knowledge, while 26.4% indicated less knowledge. Similar to the PC experiment data, the VR data were processed using the Box-Cox transformation to address non-normality. Specifically, the *λ* values for different variables are as follows: 1.097 for Extraversion, 0.626 for Agreeableness, 1.072 for Conscientiousness, 0.930 for Emotional stability, 1.059 for Openness to experiences, 1.045 for T_0_, and 1.506 for T_1_. The skewness and kurtosis of the transformed data are presented in Table 6, where the absolute values of both coefficients are less than 1.96, indicating no significant deviation from zero. This confirms that the transformed data follows a normal distribution, ensuring its suitability for further statistical analysis.

**Table 6:** Kurtosis and Skewness of VR data.

	Kurtosis	Kurtosis coefficient	Skewness	Skewness coefficient
1. Extraversion	0.020	0.078	0.355	0.695
2. Agreeableness	−0.045	−0.174	−0.970	−1.898
3. Conscientiousness	−0.019	−0.074	−0.065	−0.127
4. Emotional stability	0.040	0.155	0.639	1.250
5. Openness to experiences	0.000	0.000	−0.141	−0.276
6. T_0_	0.092	0.357	0.950	1.859
7. T_1_	−0.136	−0.527	−0.317	−0.620

To examine the relationships between personality traits and trust in the VR experiment, Pearson correlation analysis was performed. The results, presented in Table 7, reveal a positive correlation between T_0_ and both Extraversion (*r* = 0.323, *p* < 0.01) and Openness to experiences (*r* = 0.278, *p* < 0.01). Additionally, there is a significant positive correlation between T_1_ and T_0_ (*r* = 0.414, *p* < 0.001). These findings suggest that individuals with higher levels of Extraversion and Openness to Experience are more likely to exhibit higher static trust in the UAVs, and that static trust is strongly related to dynamic trust after the interaction, supporting the hypotheses that personality traits influence both static and dynamic trust.

**Table 7:** Pearson correlation analysis.

	*M*	SD	1	2	3	4	5	6
1. Extraversion	3.433	1.422						
2. Agreeableness	2.869	0.602	−0.046					
3. Conscientiousness	3.622	1.228	0.115	0.276^**^				
4. Emotional stability	3.112	0.928	0.168	0.324^**^	0.243^*^			
5. Openness to experiences	4.092	1.262	0.359^**^	0.234^*^	0.141	0.221^*^		
6. T_0_	3.395	1.281	0.323^**^	−0.056	0.018	−0.022	0.278^**^	
7. T_1_	6.931	3.191	0.025	0.014	0.055	0.054	0.114	0.414^***^

*p < 0.05, **p < 0.01, ***p < 0.001.

Stepwise regression analysis was used to identify the key personality trait predictors of static trust. Again the five dimensions of personality trait as preditor and T_0_ as the traget and the results are shown in Table 8. From the table, it can be observed that the VIF values are all less than 10, and the Durbin-Watson value is 1.696, indicating that there is no autocorrelation in the data. The model shows statistical significance (*F* = 9.917, *p* < 0.05), and the analysis reveals that Extraversion has a positive predictive effect on T_0_ (*B* = 0.291, *p* < 0.05). Extraversion explains 10.4% of the variance in T_0_ (*R*^2^ = 0.104). These findings suggest that higher Extraversion leads to higher static trust aligning with the findings from the PC experiment and supporting H1.

**Table 8:** Effect of personality traits on T_0_.

	*B*	*β*	Sig	VIF	*F*	Model sig	*R* ^2^	Durbin-Watson
Extraversion	0.291	0.323	0.002^**^	1.000	9.917	0.002^**^	0.104	1.696
Exclude variables								
Agreeableness		−0.042	0.689	1.002				
Conscientiousness		−0.019	0.852	1.013				
Emotional stability		−0.078	0.454	1.029				
Openness to experiences		0.186	0.091	1.148				

*p < 0.05, **p < 0.01, ***p < 0.001.

Further stepwise regression analysis was performed to examine how personality traits, along with T_0_, predict dynamic trust T_1._ The results shown in Table 9. The table reveals that VIFs are less than 10, and the Durbin-Watson value is 1.567, indicating no autocorrelation in the data. The model was statistically significant (*F* = 17.603, *p* < 0.05). Notably, the five dimensions of personality trait do not have a predictive effect on T_1_ (Extraversion: *p* = 0.248; Agreeableness: *p* = 0.706; Conscientiousness: *p* = 0.634; Emotional stability: *p* = 0.529; Openness to experiences: *p* = 0.990). However, T_0_ has a positive predictive effect on T_1_ (*B* = 1.032, *p* < 0.05), explaining 17.2% of the variance in dynamic trust (*R*^2^ = 0.172). These results support H2, confirming that static trust (T_0_) serves as a foundation for the development of dynamic trust (T_1_), regardless of personality traits.

**Table 9:** Effect of T_0_ on T_1_.

	B	β	sig	VIF	*F*	Model sig	*R* ^2^	Durbin-Watson
T_0_	1.032	0.414	**0.000** ^***^	1.000	17.603	**0.000** ^***^	0.172	1.567
Exclude variables								
Extraversion		−0.121	0.248	1.117				
Agreeableness		0.038	0.706	1.003				
Conscientiousness		0.047	0.634	1.000				
Emotional stability		0.063	0.529	1.000				
Openness to experiences		−0.001	0.990	1.084				

*p < 0.05, **p < 0.01, ***p < 0.001.

In addition, to analyze the effects of different modalities on Human-Machine trust, two independent samples t-tests were conducted separately T_0_ and T_1_ between the PC and VR modalities. The results are presented in Table 10. For T_0_, the PC group had a mean score of 2.557 (SD = 0.460), while the VR group had a higher mean score of 3.395 (SD = 1.281). This difference was statistically significant (*t* = −5.715, *p* < 0.01), indicating that T_0_ was significantly lower in the PC modality compared to the VR modality. Similarly, for T_1_, the mean score for the PC group was 9.696 (SD = 3.319), whereas the VR group had a lower mean of 6.931 (SD = 3.191). This difference was also statistically significant (*t* = 5.469, *p* < 0.01), suggesting that T_1_ was significantly higher in the PC modality than in the VR modality. These results suggest that T_0_ was lower in PC modality compared to VR, whereas T_1_ was higher in PC modality than in VR modality. These findings highlight the significant impact of different modalities on Human-Machine trust.

**Table 10:** The results of the independent samples t-test.

		*N*	*M*	SD	*t*
T_0_	PC	80	2.557	0.460	−5.715^***^
	VR	87	3.395	1.281	
T_1_	PC	80	9.686	3.319	5.469^***^
	VR	87	6.931	3.191	

*p < 0.05, **p < 0.01, ***p < 0.001.

## 5 Discussion

This study explored the role of personality trait and interaction modalities in shaping static and dynamic trust in HMI. The hypotheses addressed three core questions: (1) whether personality trait significantly affects static trust, (2) whether personality trait significantly affects dynamic trust, and (3) whether static trust significantly affects dynamic trust, moderated by interaction modalities. Static trust T_0_ represents participants’ initial trust in the intelligent machine, measured before interaction, while dynamic trust T_1_ reflects trust developed after completing the experimental tasks.

The results consistently demonstrated that Extraversion significantly influenced static trust across both interaction modalities supporting Hypothesis H1. In the PC experiment, Extraversion was a significant positive predictor of static trust, explaining 9.9% of the variance in T_0_. Similarly, in the VR experiment, Extraversion also positively predicted static trust, accounting for 10.4% of the variance. These findings align with prior research, (e.g., Riedl, 2022; Kim et al., 2016), which suggests that extroverted individuals are more inclined to engage in collaborative activities and exhibit greater static trust toward intelligent systems. This result highlights the importance of individual differences in shaping static trust in HMI. Extroverted individuals are more enthusiastic, proactive, and open to collaboration, making them more likely to trust intelligent machines from the outset. This finding underscores the need to consider personality traits, such as Extraversion, when designing AI-driven interfaces, as tailoring these systems to user personality traits could enhance static trust and overall usability.

The results partially support H2, showing that personality trait significantly affects dynamic trust in the PC modality but not in the VR modality. In the PC experiment, Emotional stability emerged as a significant predictor of dynamic trust, suggesting that Emotional stability individuals are more likely to sustain trust as interactions deepen. This finding aligns with prior studies (e.g., Elson, 2019), which highlight that repeated exposure to the machine may enable individuals to develop a deeper understanding of its capabilities and limitations, facilitating more accurate trust calibration. As individuals gain familiarity through continued interaction, they can better assess the machine’s reliability, predict its behavior, and adjust their trust accordingly. This process helps mitigate initial uncertainties and fosters a more stable and well-informed trust dynamic over time. Individuals with Emotional stability are more capable of rationally evaluating the performance of intelligent machines, which fosters greater trust over time. However, in the VR experiment, no significant effect of personality trait on dynamic trust was observed. This lack of significance may be attributed to the heightened cognitive load and sensory immersion associated with VR environments, which may overshadow the influence of individual personality traits (Petersen et al., 2022). These results align with Zhou et al. (2020), who found that immersive environments can dilute the effects of personality trait on trust development. Future research could investigate whether specific features of immersive technologies moderate the relationship between personality trait and dynamic trust.

The results strongly support H3, demonstrating that static trust is a significant predictor of dynamic trust (T_1_) across both interaction modalities. In the PC experiment, T_0_ significantly influenced T_1_, explaining 21.9% of the variance in dynamic trust. Similarly, in the VR experiment, T_0_ was also a strong positive predictor of T_1_, accounting for 17.2% of the variance. These findings are consistent with prior research (e.g., Martinelli Watanuki and de Oliveira Moraes, 2024; Glikson and Woolley, 2020), which highlights the foundational role of static trust in fostering dynamic trust over time. The comparison between the PC and VR experiments reveals a moderating effect of interaction modalities. While static trust had a significant impact on dynamic trust in both modalities, the magnitude of the effect was greater in the VR modality. This difference suggests that the immersive nature of VR environments may amplify the role of static trust, as participants rely more heavily on their first impressions to navigate the complex, sensory-rich environment.

The findings of this study emphasize the importance of tailoring AI-driven interfaces to individual personality traits to foster static and dynamic trust. Combining results of the PC experiment (Table 4) and the VR experiment (Table 8), personality personality significantly affects static trust, with Extraversion showing a positive and significant effect. As Extraversion levels increase, static trust also increases. This finding highlights the importance of considering individual differences, particularly Extraversion, when designing AI-driven interfaces across platforms. Users with high Extraversion may be more inclined to engage with and use these interfaces. Tailoring interface designs to users’ personality traits can enhance personalized experiences. The results also demonstrate that static trust significantly predicts dynamic trust across both PC and VR modalities (Tables 5, 9). This highlights the importance of fostering static trust to encourage sustained engagement. For instance, AI-driven interfaces should prioritize creating positive first impressions through user-friendly designs, intuitive interactions, and introductory tutorials for novice users. Such strategies can help establish static trust, thereby enhancing dynamic trust and improving user retention. Furthermore, the findings suggest that Emotional stability positively influences dynamic trust in the PC modality, with more emotionally stable individuals showing a greater ability to sustain trust over time. Designing interfaces that provide consistent feedback, reduce stress, and facilitate smooth interactions may help users with lower Emotional stability to maintain trust. While this study highlights the significance of personality traits, it also underscores the necessity of platform-specific considerations in interface design to ensure effective HMI.

The lack of a significant effect of personality trait on dynamic trust in the VR experiment results aligns with Zhou et al.’s findings (Zhou et al., 2020), indicating that immersive environments introduce complexities that may overshadow personality trait influences. Factors such as cognitive load and uncertainty inherent in VR modality could moderate the relationship between personality traits and trust development (Petersen et al., 2022). Future research should examine how these contextual factors interact with personality trait to influence trust, especially under varying levels of task complexity and immersion. Additionally, the nuanced effects of personality trait on trust suggest that static personality trait measures may not fully capture dynamic trust behaviors. For example, Zhou et al. (2020) classified participants’ personality traits into high and low levels using a seven-point Likert scale, revealing more granular differences in trust development. In this study, Table 11 highlights significant differences in trust dynamics across personality trait levels. For instance, individuals with low Extraversion exhibited significant differences between static and dynamic trust (Z = −3.558, *p* < 0.001), likely due to their reserved and cautious nature (Ahmad et al., 2021). Similarly, significant differences were observed in Agreeableness, Conscientiousness, Emotional stability, and Openness to Experience, with effects varying based on high and low levels of each personality trait. These findings indicate that personality trait’s impact on trust development is context-specific and may depend on both personality trait levels and contextual factors. Moreover, individuals with high Conscientiousness often view technology as useful, enabling them to engage more effectively with intelligent systems (Mouakket, 2018). Similarly, users with high Openness to Experience are more likely to adopt new technologies, explore novel concepts, and demonstrate greater willingness to collaborate with intelligent systems (Svendsen et al., 2013). These insights suggest that future studies should adopt more dynamic personality trait measures and explore the interactions between specific personality trait levels and immersive environments. Finally, while this study provides valuable insights into personality trait and trust dynamics, additional research is needed to examine how personality trait dimensions interact with contextual factors such as cognitive load, immersion, and task complexity in immersive environments. Employing experimental designs that isolate these factors could yield a deeper understanding of how to optimize trust-building strategies in diverse HMI scenarios.

**Table 11:** Comparison of T_0_ and T_1_ at different trait levels.

	Level	*Z*-value	Sig
Extraversion	High	−1.921	0.055
	Low	−3.558	0.000^***^
Agreeableness	High	−2.978	0.003^**^
	Low	−2.946	0.003^**^
Conscientiousness	High	−3.776	0.000^***^
	Low	−1.816	0.069
Emotional stability	High	−3.699	0.000^***^
	Low	−2.231	0.026^*^
Openness to experiences	High	−3.651	0.000^***^
	Low	−1.936	0.053

*p < 0.05, **p < 0.01, ***p < 0.001.

## 6 Conclusion

This study contributes to the growing body of research on Human-Machine trust by exploring the impact of personality trait on both static and dynamic trust across two distinct interaction modalities: PC and VR. The novelty of this research lies in its examination of how personality traits influence trust development in HMI across both traditional and immersive environments. By investigating the relationship between static and dynamic trust, this study offers a comprehensive understanding of how static trust shapes trust evolution in both PC and VR contexts. The experimental findings show that on the PC modality, Extraversion significantly influences both static and dynamic trust, with more extroverted individuals displaying higher levels of static trust and sustaining that trust throughout the interaction. Furthermore, Emotional stability was found to significantly influence dynamic trust, suggesting that emotionally stable individuals are more likely to develop and maintain trust over time. On the VR modality, while personality trait still significantly influenced static trust, the effect of personality trait on dynamic trust was not observed. However, the strong relationship between static trust and dynamic trust in VR mirrors the results on the PC modality, highlighting the foundational role of static trust in fostering ongoing trust in immersive environments. This study’s findings have important implications for the design of AI-driven interfaces. Personality traits, especially Extraversion, should be considered when designing user-centered systems, as individuals with higher Extraversion may engage more readily with intelligent machines, particularly in immersive environments. Additionally, the results underscore the importance of fostering static trust through intuitive and user-friendly designs, as this trust significantly influences dynamic trust, particularly in long-term interactions.

However, this study has several limitations. First, the sample was primarily composed of Chinese students, which limits the cross-cultural applicability of the findings. Future research should aim to include more diverse cultural backgrounds to explore how cultural differences influence trust development in HMI. Second, Disparities in demographic composition between experimental groups pose a potential threat to internal validity, particularly when there is a significant imbalance in gender distribution among participants. Such differences may introduce unintended biases, potentially influencing the reliability and generalizability of the study’s findings, a larger and more diverse sample would increase the generalizability of the results. In this study, trust was measured using a single-question approach, which presents certain limitations, as the obtained values may not accurately reflect the participants’ actual level of trust. Future research should adopt a more specialized Human-Machine trust scale to ensure a more precise and comprehensive assessment of trust. Lastly, this study focused on two phases of trust development (static and dynamic trust); future research could investigate additional phases of trust development, such as maintenance and erosion, to provide a more comprehensive understanding of the longitudinal dynamics of trust in HMI.

Future research should also explore how different personality traits interact with various contextual factors, such as cognitive load, immersion, and task complexity, especially in immersive environments like VR. Additionally, investigating how personality traits influence trust development over longer periods or multiple phases of interaction would offer valuable insights into the sustainability of trust in HMI. Finally, exploring cross-platform interactions, where users switch between modalities (e.g., from PC to VR), could provide a deeper understanding of how trust transfers across different interaction contexts. By addressing these limitations and expanding the scope of future studies, we can develop a more nuanced understanding of the complex relationship between personality trait, interaction modalities, and Human-Machine trust, leading to more effective and personalized AI-driven systems.

## Data Availability

The raw data supporting the conclusions of this article will be made available by the authors, without undue reservation.

## Footnotes

^1^ https://openai.com/chatgpt/overview/

^2^ https://yiyan.baidu.com/

^3^ https://tongyi.aliyun.com/qianwen/

^1^ https://www.picoxr.com/cn
